# Mapping wrist motion: 3D CT analysis after scapholunate ligament transection

**DOI:** 10.1111/joa.14119

**Published:** 2024-08-02

**Authors:** Dominik Promny, Dominik Gill, Stefan Lyer, Christoph Alexiou, Thomas Buder, Winfried Neuhuber, Raymund E. Horch, Andreas Arkudas

**Affiliations:** ^1^ Department of Plastic and Hand Surgery, Laboratory for Tissue Engineering and Regenerative Medicine, Friedrich‐Alexander‐Universität Erlangen‐Nuernberg FAU Universitätsklinikum Erlangen Erlangen Germany; ^2^ Department of Otorhinolaryngology, Head & Neck Surgery, Section of Experimental Oncology & Nanomedicine (SEON), Professorship for AI‐Controlled Nanomaterials, Friedrich‐Alexander‐Universität Erlangen‐Nuernberg FAU Universitätsklinikum Erlangen Erlangen Germany; ^3^ Institute of Anatomy, Department I Friedrich‐Alexander‐Universität Erlangen‐Nuernberg FAU Erlangen Germany

**Keywords:** carpal bone motion, CT imaging analysis, scapholunate (SL) ligament injury, wrist biomechanics, wrist kinematics

## Abstract

The injury of the scapholunate (SL) ligament is common in wrist traumas leading to pain and reduced wrist function. The wrist's unique joint design and possible underlying theories as the carpal row theory were subject to earlier investigations studying wrist kinematics. Nevertheless, a comprehensive understanding of how SL ligament injuries affect wrist biomechanics is still lacking. Through a quantitative analysis of carpal bone motion patterns, we evaluated the impact on wrist kinematics occurring after SL ligament injury. We conducted a study using computer tomography imaging to analyse wrist kinematics after SL ligament transection in 21 fresh‐frozen anatomical specimens. The collected data were then transformed into 3D models, employing both standardized global and object coordinate systems. The study encompassed the evaluation of rotation and translation for each individual carpal bone, as well as the ulna, and all metacarpal bones in reference to the radius. The study showed a significant increase in rotation towards palmar (*p* < 0.01), particularly notable for the scaphoid, following transection of the SL ligament during palmar flexion. Ulnar deviation did not significantly affect rotation or translation, and radial deviation also showed no significant changes in rotation or translation. The study highlights the significance of the SL ligament in wrist kinematics, revealing that SL ligament tears lead to changes in wrist motion. While we observed significant rotational changes for the scaphoid, other carpal bones showed less pronounced alterations, emphasizing the complexity of wrist biomechanics.

## Graphical abstract

The study investigates the impact of scapholunate (SL) ligament injury on wrist kinematics using computer tomography imaging and 3D modelling. Findings reveal significant rotational changes, particularly for the scaphoid during palmar flexion, underscoring the SL ligament's critical role in wrist biomechanics.

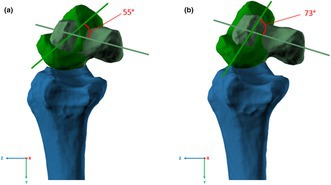

## 1 INTRODUCTION

The scapholunate (SL) ligament is an intrinsic ligament, which primarily stabilizes the SL joint (Rajan & Day, 2015a; Sokolow & Saffar, 2001; Taleisnik, 1976). Its injury is common in wrist trauma, often resulting from a fall on an outstretched hand in isolation or in association with a distal radius fracture (Guss et al., 2015; Majima et al., 2008; Mayfield, 1980).

Patients with this injury often experience pain, limited range of motion and weakened grip strength, impacting daily activities (Athlani et al., 2019; Grüner et al., 2023). Clinical examination may elicit discomfort with palpation and the Watson scaphoid shift test.

The wrist features dual joints—the radiocarpal and midcarpal—stabilizing motion up to 180° of range from full wrist flexion to extension, with diverse kinematic concepts explored historically. Besides the columnar theory, published by Navarro (1921) and edited by Taleisnik (1976), the carpal row theory is based on the idea of a proximal and distal arranged row of the carpal bones, which was first published by Johnston (1907). The carpal row theory states that the main wrist's movement takes place between the two rows instead of the single carpal bones themselves. The findings of our previous investigations of wrist kinematics, published by Gill et al. (2022), support the row theory. The observations showed that the majority of motion occurs between the carpal rows, and furthermore, presenting a special role for the scaphoid and the first metacarpal bone.

The long‐term impact on the biomechanical and kinematic conditions of the SL ligament injury can lead to progressive arthritic changes and irreversible wrist damage (Larsen et al., 1995; Orr et al., 2010), emphasizing the need for early diagnosis and treatment to restore stability and prevent arthritis.

However, the interplay of the single wrist bones and their ligamentous connections resulting in the above‐mentioned symptoms and biomechanical changes is yet not fully understood. To better comprehend the impact of an SL transection on the wrist kinematics, we conducted an extensive study to investigate the biomechanics of the singular carpal bones and the adjoining metacarpals, the ulna and the radius. Therefore, we analysed the rotation and translation in a three‐dimensional model using wrist specimens with transacted SL ligament and computer tomography (CT) imaging.

## 2 MATERIALS AND METHODS

### 2.1 Anatomical specimens

The research was conducted on 21 fresh‐frozen anatomical specimens regarding the Institute's body donation program for didactic, research and postgraduate training purposes. For the investigations we used upper extremity specimens including wrists and hands without prior surgery and carpal ligaments were intact. X‐ray diagnostics were conducted to identify wrist pathologies in general and especially for detecting any indications of an SL ligament injury, such as dissociation between the scaphoid and lunate. If any indication of an SL ligament injury was identified, the specimen was excluded. Two specimens were replaced due to arthrosis and strong osteoporosis in the screening X‐ray diagnostics. Data were then collected with the wrist intact and after isolated transection of the complete SL ligament. Motion analysis included carpal bones, metacarpals and ulna movement relative to the radius during wrist flexion, extension, ulnar and radial deviation.

### 2.2 Imaging/diagnostics

The technical details of image acquisition, image analysis and the determination of the applied coordinate systems and transformation matrices, have been previously described in detail by Gill et al. (2022). The following paragraphs briefly present the underlying techniques.

**FIGURE 1:**
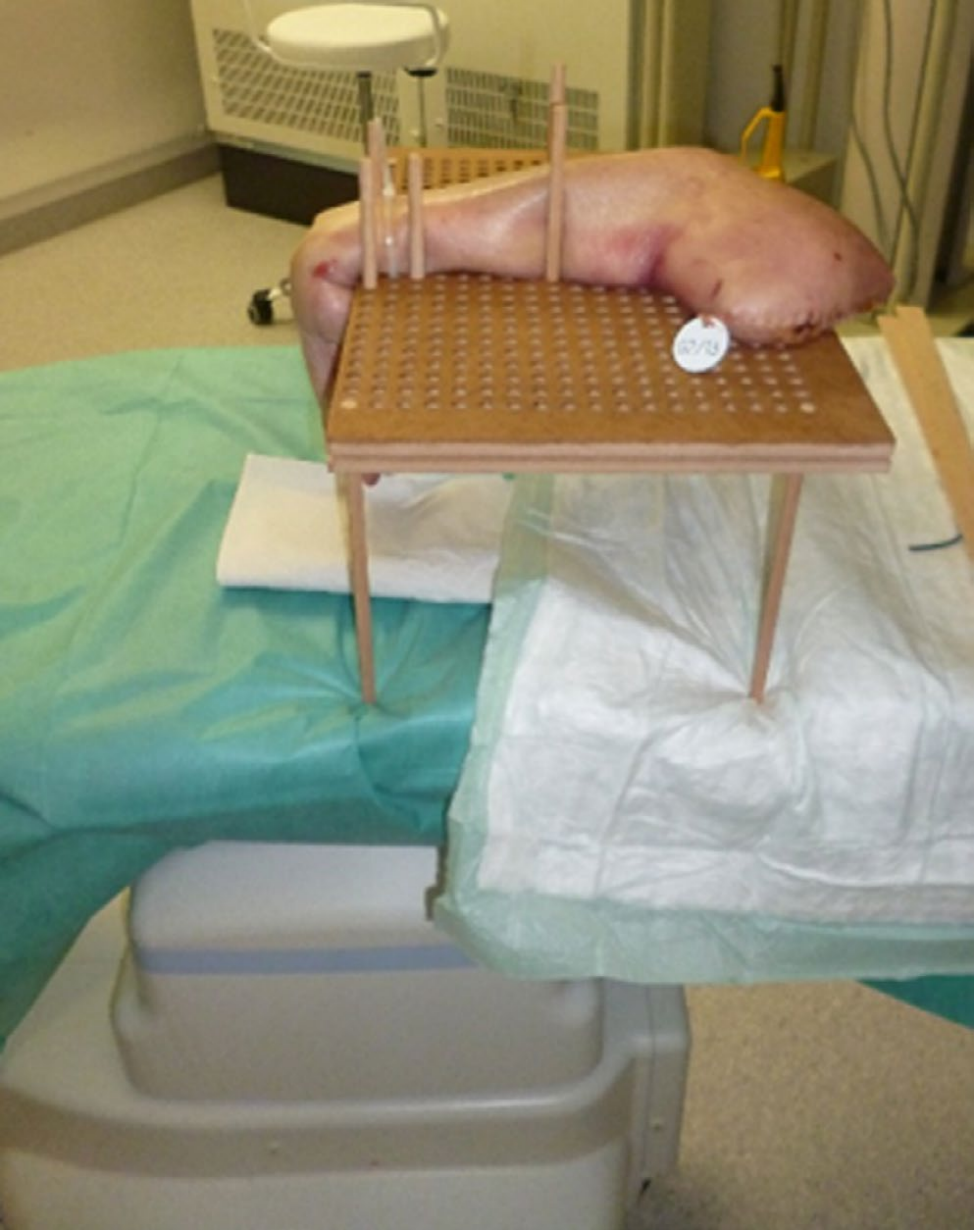
Experimental setup with immobilized specimens on a wooden structure inside a CT scanner. To fixate extension strings were employed to stabilize the specimens on the wooden contraption. CT, computer tomography.

CT scans were performed in a Siemens Healthineer Artis Zee (Siemens Healthcare GmbH, Erlangen, Germany) at 55 kV, 75 mAS, 20 s scan time and 0.5 mm slice thickness. To avoid interfering metal artefacts a wooden contraption was applied for the correct positioning of the specimens during the CT scan (see Figure 1). Specimens were positioned in neutral position, flexion, extension, radial deviation and ulna deviation using wooden sticks and strings for fixation. The particular motion was consistently adjusted to its maximal extent.

A total of 105 CT scans resulted as for each specimen after SL ligament transection five CT scans in the respective position were performed. The results of initial CT scans for the intact wrist were detailed by Gill et al. (2022).

**FIGURE 2:**
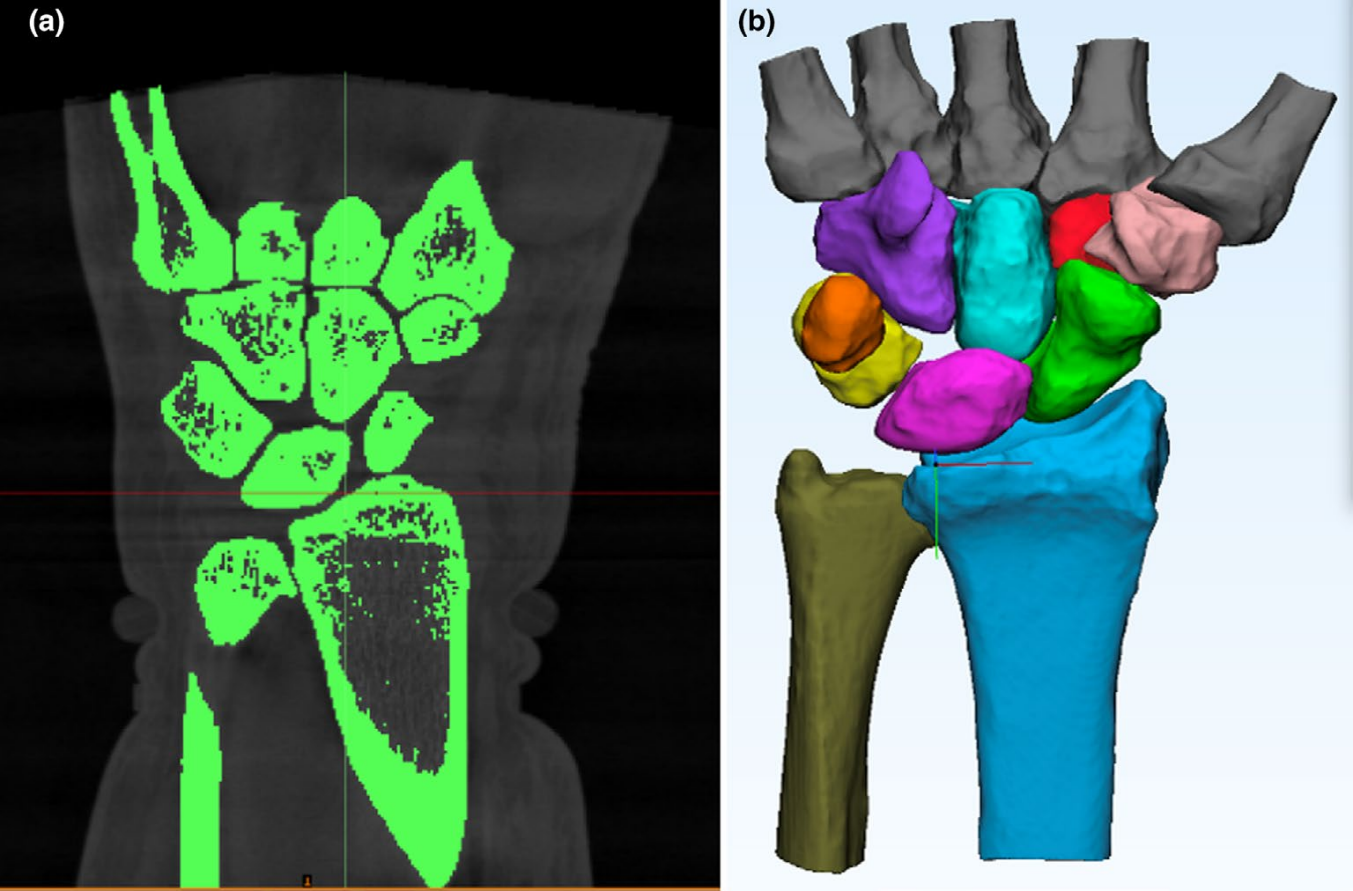
(a) The CT scans were processed to generate a 3D model by segmenting each individual bone. This involved the selection of individual bones, ensuring unambiguous identification within the program. This process was repeated for each bone to obtain a comprehensive 3D model of the wrist. (b) Subsequently, post‐segmentation tools such as smoothing and wrapping were employed to enhance the surface details. CT, computer tomography.

Images were exported to digital imaging and communications in medicine (DICOM) files and analysed using Mimics® software by Materialise (Materialise NV, Leuven, Belgium). Semi‐automatic segmentation techniques based on Hounsfield units were applied to reconstruct all eight carpal bones, the five metacarpals, the radius and ulna (see Figure 2). Subsequently, the three‐dimensional surface data was transferred to 3‐Matic® software by Materialise for refinement, employing post‐segmentation tools such as wrapping and smoothing.

### 2.3 Data analysis

To analyse bone movement, coordinate system determination and transformation matrix analysis are essential.

**FIGURE 3:**
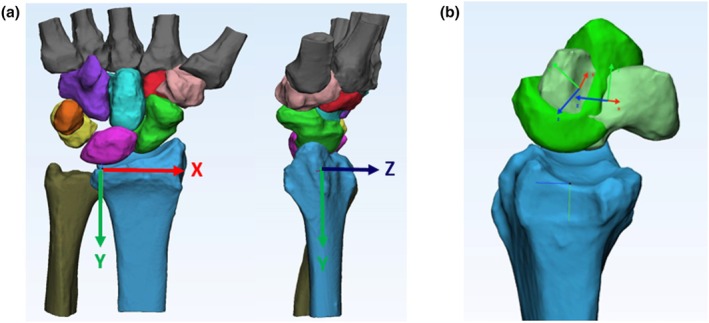
(a) In the radius, a referential coordinate system is established using the midpoint between the palmar and dorsal edges of the distal radius, positioned at the ulnar notch. Additionally, an object‐related coordinate system is created within each bone, aligning it with an automatic object coordinate system. (b) This alignment is achieved by using the mass centres of each bone's surface as the origin and considering the three inertia axes of these surfaces under motion. Here the movement of the scaphoid is depicted in neutral position and in flexion of the wrist.

In order to ensure the precision of the virtual alignment process, we adopted a meticulous methodology that integrated both global and object‐specific coordinate systems. Initially, semi‐automatic segmentation techniques based on Hounsfield units were utilized to reconstruct all carpal bones and adjoining structures from CT scans. For alignment, the midpoints between the palmar and dorsal edges of the distal radius at the ulnar notch were defined as the origin, providing a consistent reference point (see Figure 3) (DiBenedetto et al., 1991; Orr et al., 2010). The radii were cropped to 60 mm proximal to the radial styloid process to determine their volumetric centre for the coordinate systems. The XY‐plane was defined by the volumetric centre, midpoint of the ulnar notch, and longitudinal inertia axis, with the *X*‐axis radial and the *Y*‐axis proximal. The *Z*‐axis is extended dorsally perpendicular to the XY‐plane. This method was selected to achieve an optimal balance between precision and practicality.

To validate the accuracy of our alignment process, we conducted repeated trials and compared the resultant alignments for consistency. Additionally, the transformations were cross‐verified against the CT scans to ensure that the virtual models accurately represented the anatomical structures.

The flexion and extension described a rotation around the *X*‐axis, the radial and ulnar deviation defined a rotation around the *Z*‐axis, and the pronation and supination were depicted by a rotation around the *Y*‐axis. All surfaces of the remaining bones were aligned to an automatic object coordinate system, which used the volumetric centres of each surface as the origin and the three inertia axes of these surfaces. The direction of these inertia *XYZ*‐axes is continuously located in the same direction automatically.

The transformation matrix depicts bone movement relative to its neutral position, defining rotation and translation compared to the reference position of each bone. The radii were aligned with the reference radius of the hand in neutral position, maintaining original relative positions. This allowed assessment of bone kinematics in four positions relative to the neutral position in the referential coordinate system. Finally, values for rotations and translations were cross‐checked with CT scans for verification.

### 2.4 Statistical analysis

We used GraphPad Prism® version 9.5.1 (GraphPad Software, La Jolla, CA, USA) for descriptive statistics. Following a Shapiro–Wilk test on normal distribution, a two‐way analysis of variance for parametric data and a Kruskal‐Wallis test for nonparametric data were conducted. Pairwise multiple comparisons with Tukey's or Dunn's correction were applied if significant. A *p*‐value ≤ 0.05 was considered to indicate a significant difference.

## 3 RESULTS

The study included 21 wrists with a complete SL ligament transection, 10 from females (mean age 80.8 years) and 11 from males (mean age 77.5 years). To facilitate comprehension, the results for rotation and translation movements are separately presented for each action (flexion, extension, ulnar deviation and radial deviation) compared to the neutral position.

### 3.1 Flexion

**FIGURE 4:**
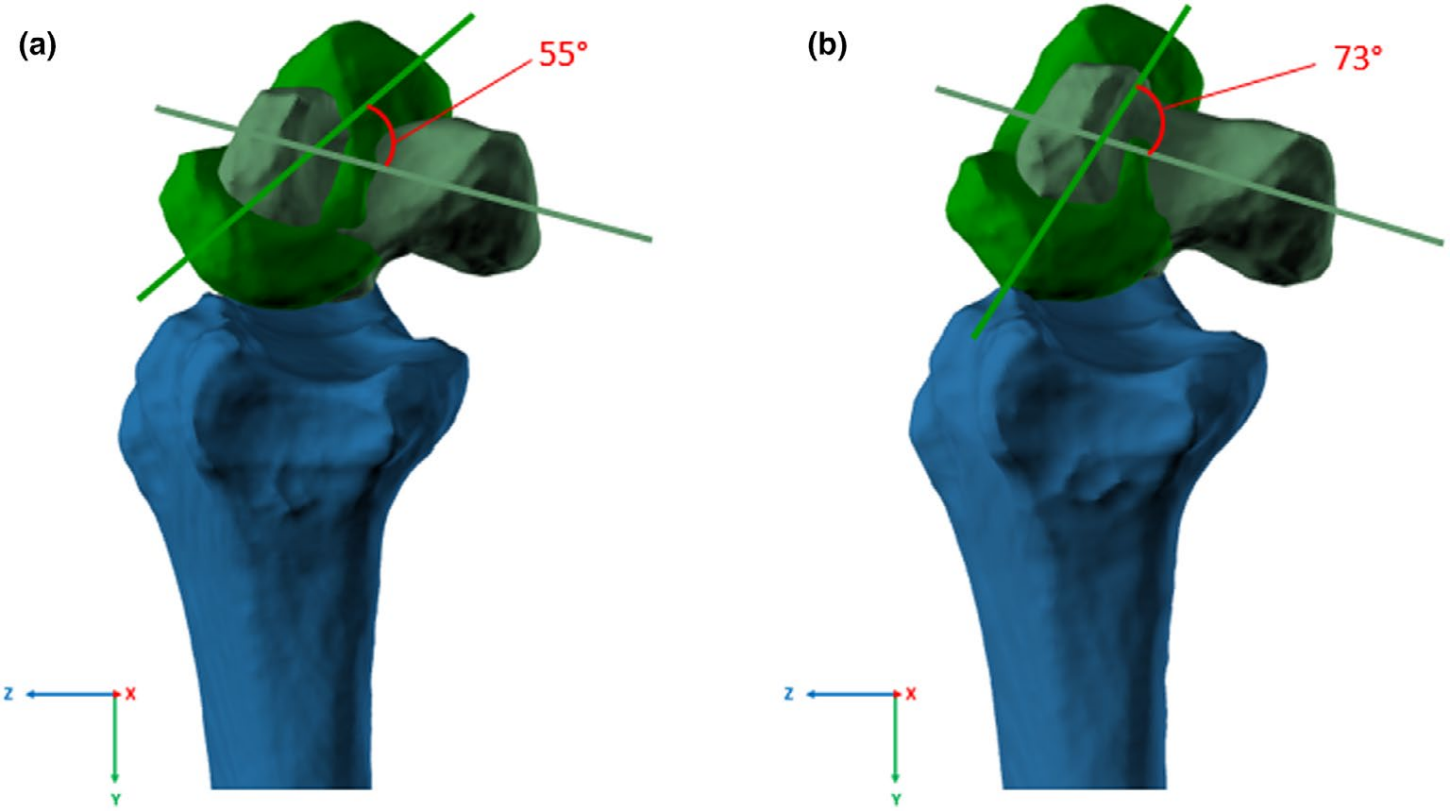
Exemplary 3D rotation analysis for the scaphoid in neutral position (light green) and flexion (muted green) with intact SL ligament (a) and transacted SL ligament (b). SL, scapholunate.

The analysis showed that during flexion the main rotation of all bones occurred in the *x*‐axis applying to both the uninjured wrist as well as the wrist with transacted SL ligament (see Figure 4). After the SL ligament transection all bones besides the ulna rotate to a greater extent in comparison to motion with intact SL ligament in the *X*‐axis and except for metacarpal 4 in the *Z*‐axis. In the *X*‐axis analysis of the proximal row, the scaphoid showed significantly the greatest rotation increase after SL ligament transection with a mean difference of 11.71° (*p* < 0.01).

With an intact SL ligament, the scaphoid demonstrates already significant rotation in the analysis of the carpal bones' relation to the lunate, triquetrum and pisiform (*p* < 0.0001). However, upon transection of the SL ligament, the scaphoid exhibits an even more pronounced degree of rotation compared to the other bones of the proximal row (*p* < 0.001).

**FIGURE 5:**
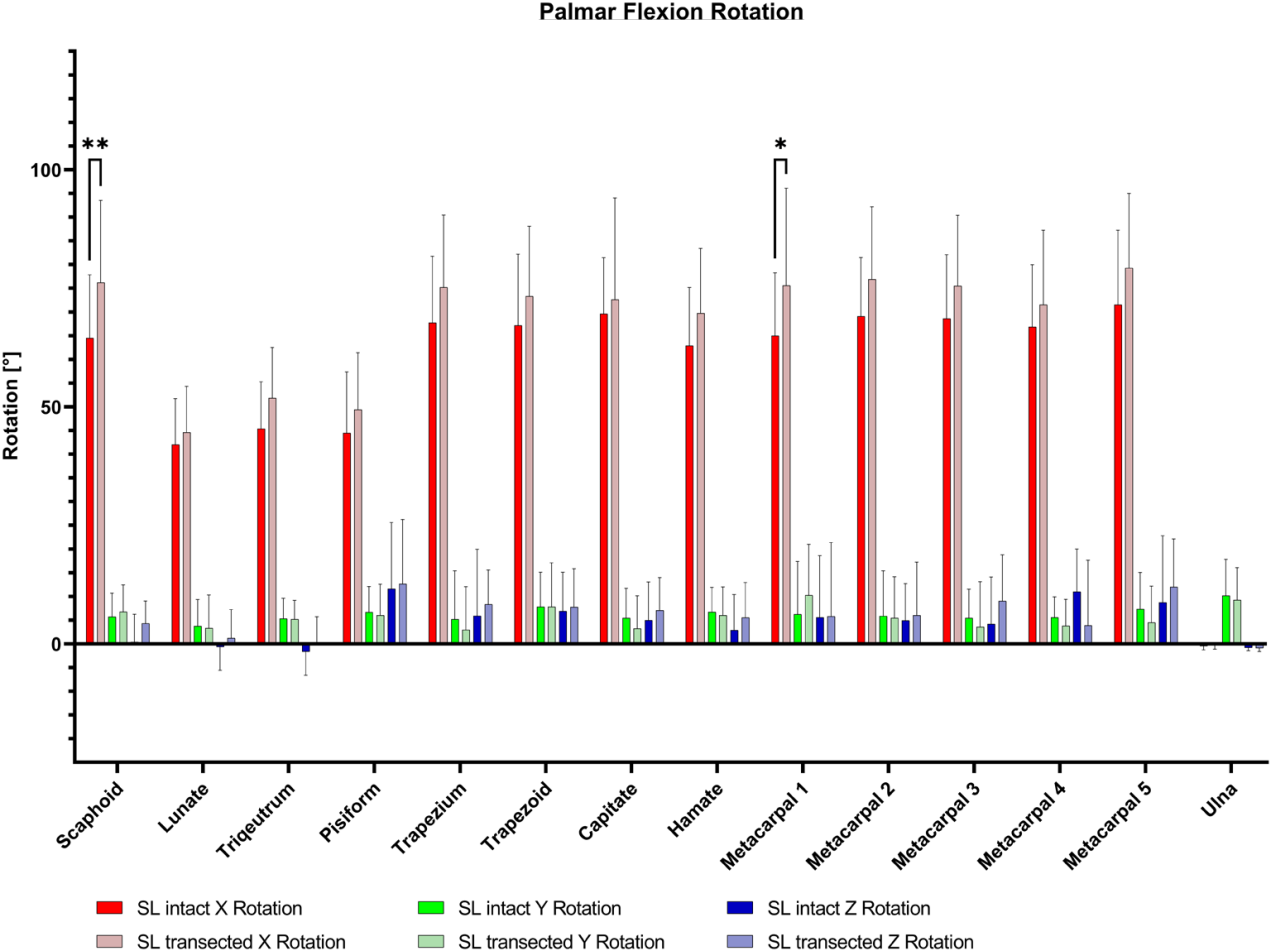
Following the SL transection, all bones except the ulna exhibit a more substantial rotation in the *X*‐axis, and with the exception of metacarpal 4, there is an increased rotation in the *Z*‐axis compared to the intact SL ligament condition. In the *X*‐axis analysis of the proximal row, the scaphoid demonstrates the most significant increase in rotation after the SL ligament transecvtion. SL, scapholunate. *p < 0.05; **p < 0.01.

Furthermore, there is a significant increase in the rotational movement of the first metacarpal bone following the transection of the SL ligament (*p* < 0.05) (see Figure 5).

**FIGURE 6:**
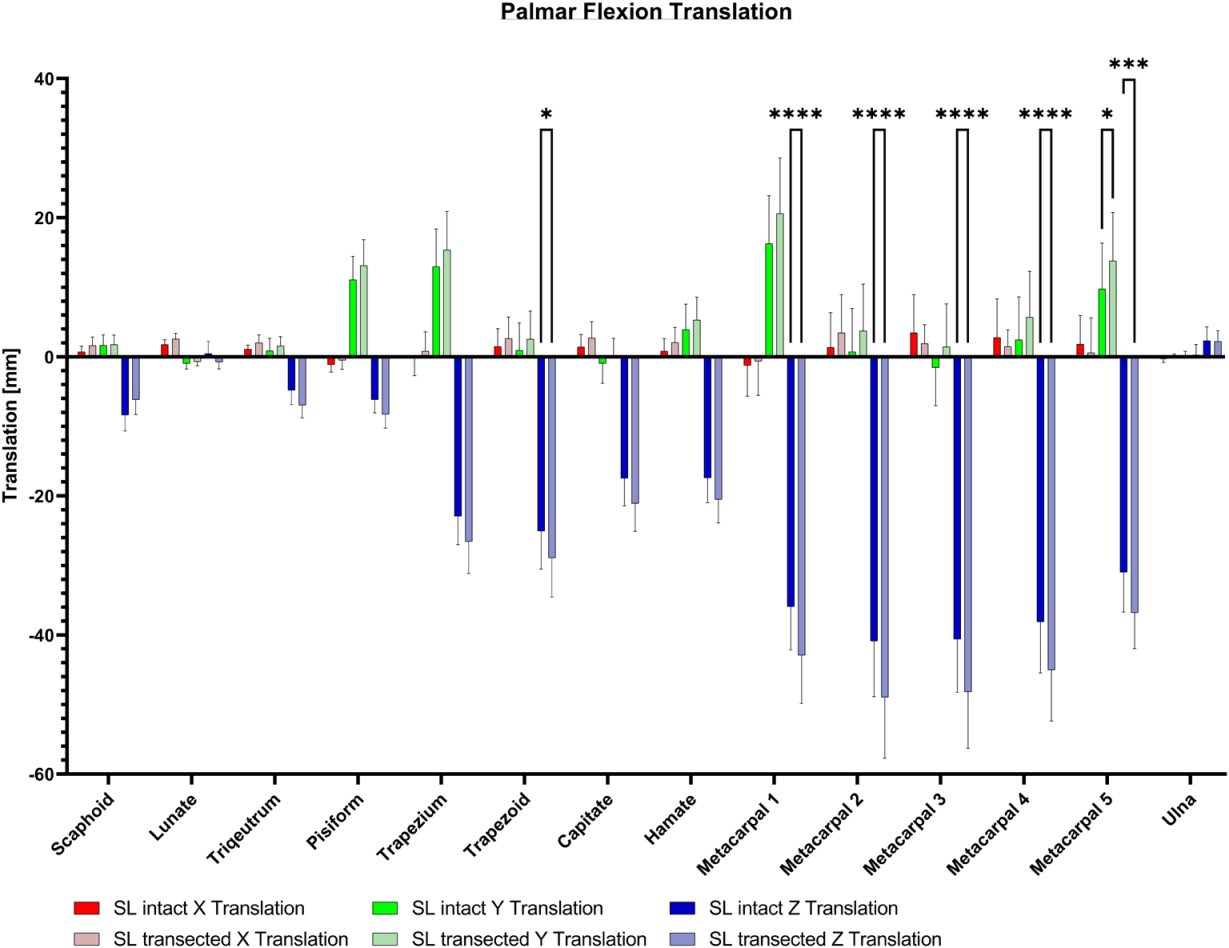
The *Z*‐axis displayed the most pronounced translation, escalating with a more distal location of the carpal bones. After the SL ligament transection, a significant translation toward palmar was observed for the trapezoid and metacarpal bones (*p* < 0.05–0.0001). Additionally, following the SL ligament transection, the metacarpal 5 translated significantly more toward proximal (*p* < 0.05). SL, scapholunate. *p < 0.05; ***p < 0.001; ****p < 0.0001.

**FIGURE 7:**
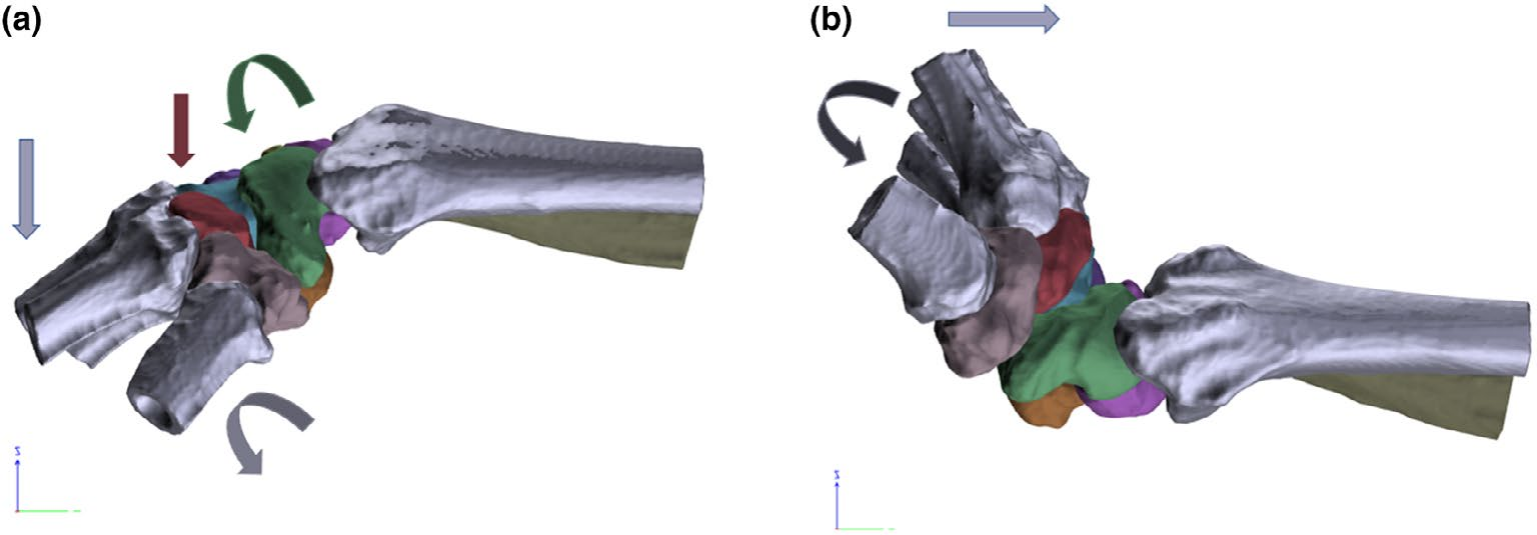
(a) Following the transection of the SL ligament, a notable increase in rotation was observed in the scaphoid (green curved arrow) and first metacarpal (gray curved arrow) during palmar flexion. Additionally, all metacarpals (grey straight arrow) and the trapezoid (red straight arrow) exhibited a significant translational movement toward palmar. (b) Dorsal extension resulted in a significant proximal motion for metacarpals 2–4 (light grey straight arrow) and a significant rotation increase for the 5th metacarpal (dark grey curved arrow). SL, scapholunate.

The translation assessment revealed that the *Z*‐axis exhibited the most substantial translation, which intensified with a more distal localization of the carpal bones (see Figure 6). Particularly, following the transection of the SL ligament, there was a significant translation increase toward palmar for the trapezoid and metacarpal bones (*p* < 0.05–0.0001) (see Figure 7). The analysis of the translation among individual carpal bones showed a significant increase after the transection of the SL ligament, with an enhanced translation of individual wrist bones in relation to the scaphoid in the *Z*‐axis analysis. (*p* < 0.01). Additionally, in both the *Z*‐axis and *Y*‐axis, the proximal row demonstrates significantly less translation compared to all the metacarpal bones (*p* < 0.05–0.001). Furthermore, the *Y*‐axis analysis revealed a significant increase in the translation toward proximal of the metacarpal 5 after SL ligament transection (*p* < 0.05).

### 3.2 Extension

**FIGURE 8:**
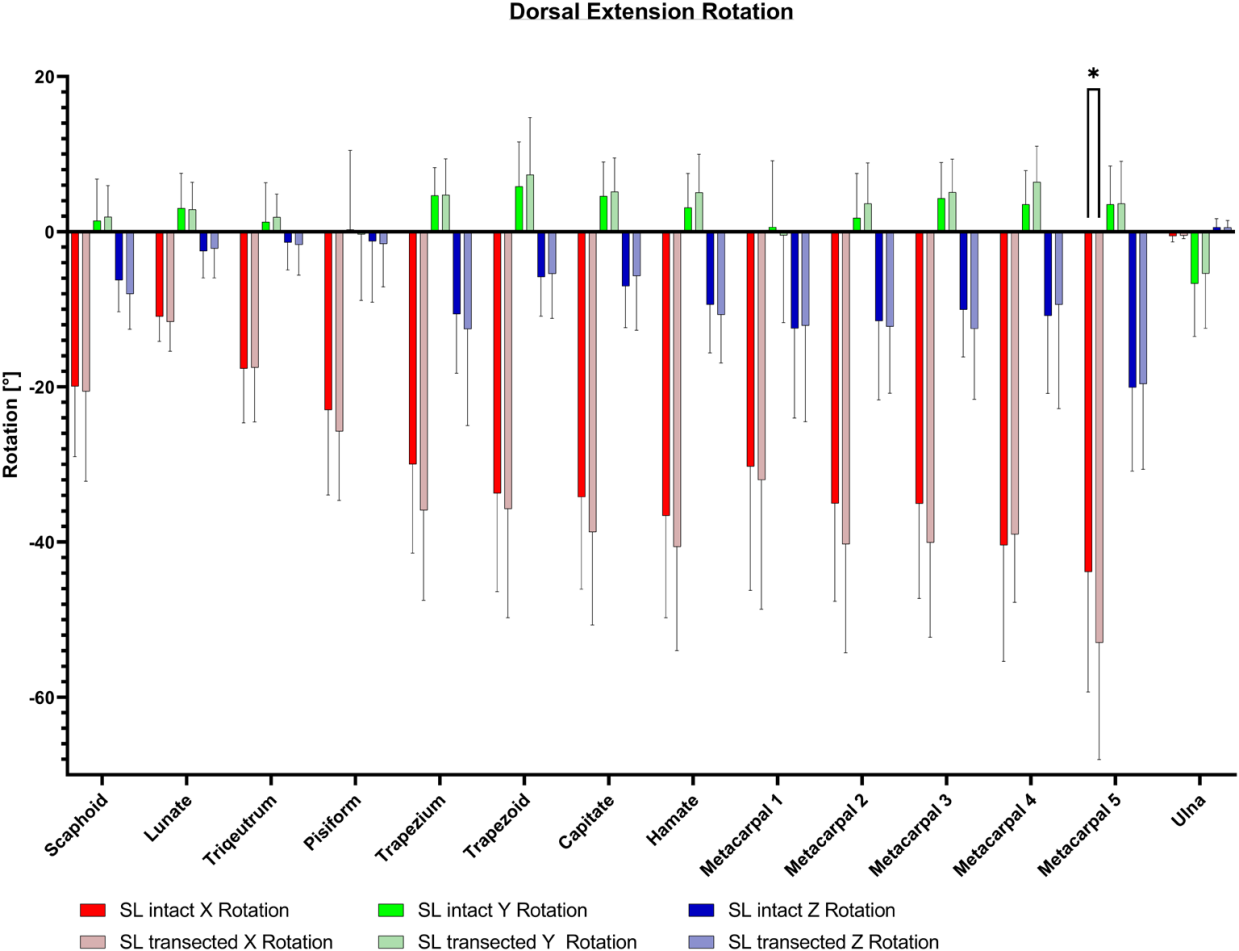
The analysis of the rotation findings in extension resulted in rotation increase being only significant for the fifth metacarpal (*p* < 0.05). Further examination revealed notable extension in the bones of the distal row, with an even more pronounced extension observed in the second to fifth metacarpals in the *X*‐axis analysis. *p < 0.05.

In terms of rotation during extension, the analysis revealed a pronounced extension for the bones of the distal row, with even greater extension observed for the second to fifth metacarpals in the *X*‐axis analysis (see Figure 8). In contrast, the first metacarpal bone showed a trend for a smaller rotation than the distal row. The transection of the SL ligament resulted in partial rotation increases being only significant for the fifth metacarpal with a mean difference of 9.13° (*p* < 0.05).

**FIGURE 9:**
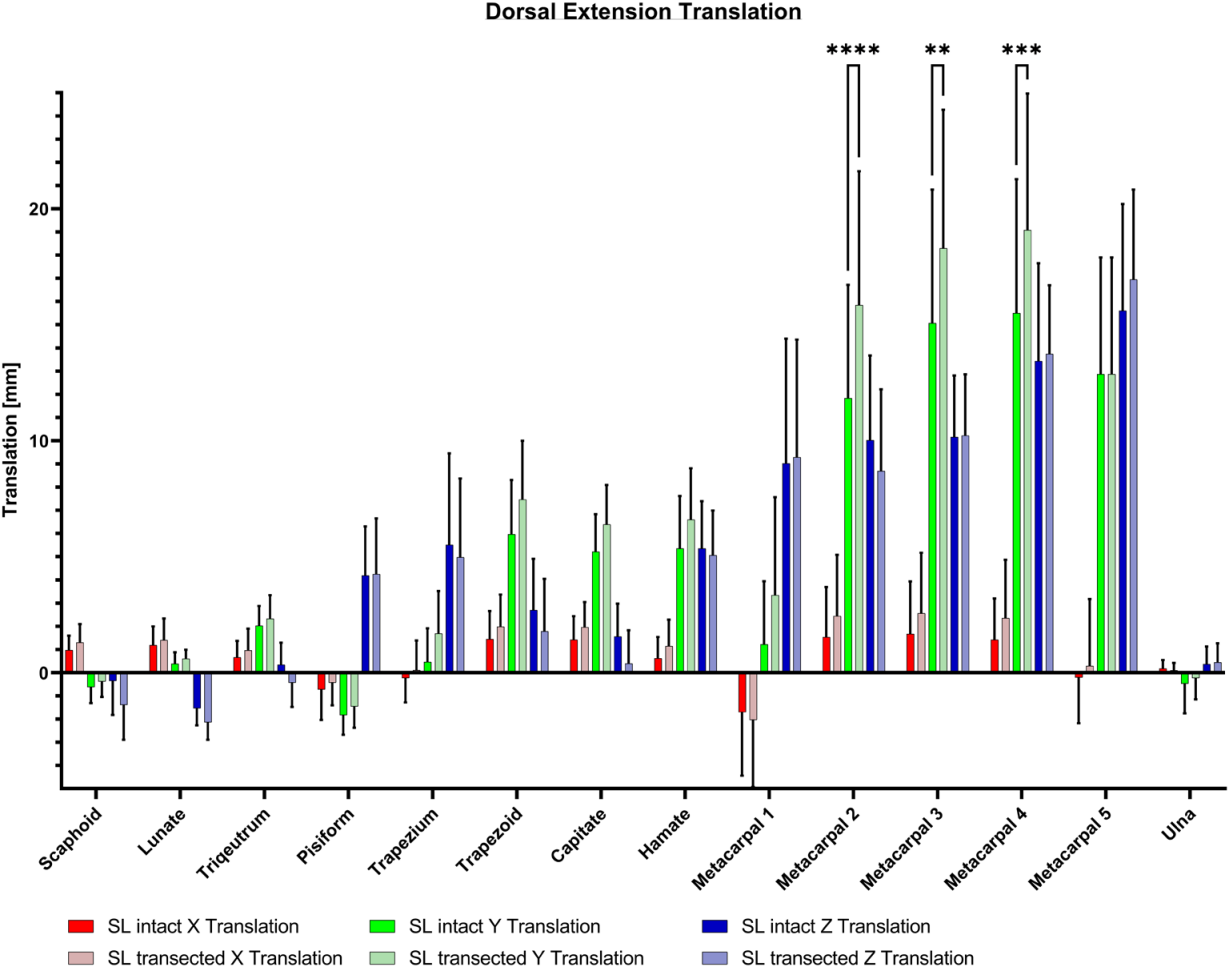
Following the transection of the SL ligament, a significant increase in translation along the *Y*‐axis was observed for the second, third and fourth metacarpal bones during wrist extension (*p* < 0.01–0.001). In contrast, both the proximal and distal row showed minimal changes, with results indicating no statistical significance. SL, scapholunate. **p < 0.01; ***p < 0.001; ****p < 0.0001.

In the translation analysis for extension, there was a notable increase in translation after SL ligament transection along the *Y*‐axis towards proximally for the second, third and fourth metacarpal bones, showing statistical significance (*p* < 0.01–0.001) (see Figure 7). The proximal and distal rows experienced only slight impacts, with results indicating no statistical significance (see Figure 9).

Interestingly, the first metacarpal bone exhibited a translation in the opposite direction, towards ulnar, during the *X*‐axis analysis compared to the other metacarpal bones, showing a radial translation. The transection of the SL ligament did not alter the translation direction of the first metacarpal bone. The translation of the first metacarpal bone was found to be significant before and after SL ligament transection towards nearly all carpal bones of both the proximal and distal rows (*p* < 0.05–0.001), except for the motion towards the pisiform and trapezium, which did not reach significance.

### 3.3 Ulnar deviation

In the analysis of bone rotation following ulnar deviation, the influence of SL ligament transection did not show any significant impact on the carpal bones subsequent to SL ligament transection. The *Z*‐axis demonstrated the greatest range of motion for all bones with a motion towards ulnar. This movement was particularly pronounced in the metacarpals and the bones of the distal row. In ulnar deviation, the bones of the proximal row exhibit a rotation towards dorsal opposite to that of the distal row and the metacarpals in the *X*‐axis analysis, being significant for the bones of the proximal row in relation to trapezium, trapezoid and capitate (*p* < 0.01–0.001).

**FIGURE 10:**
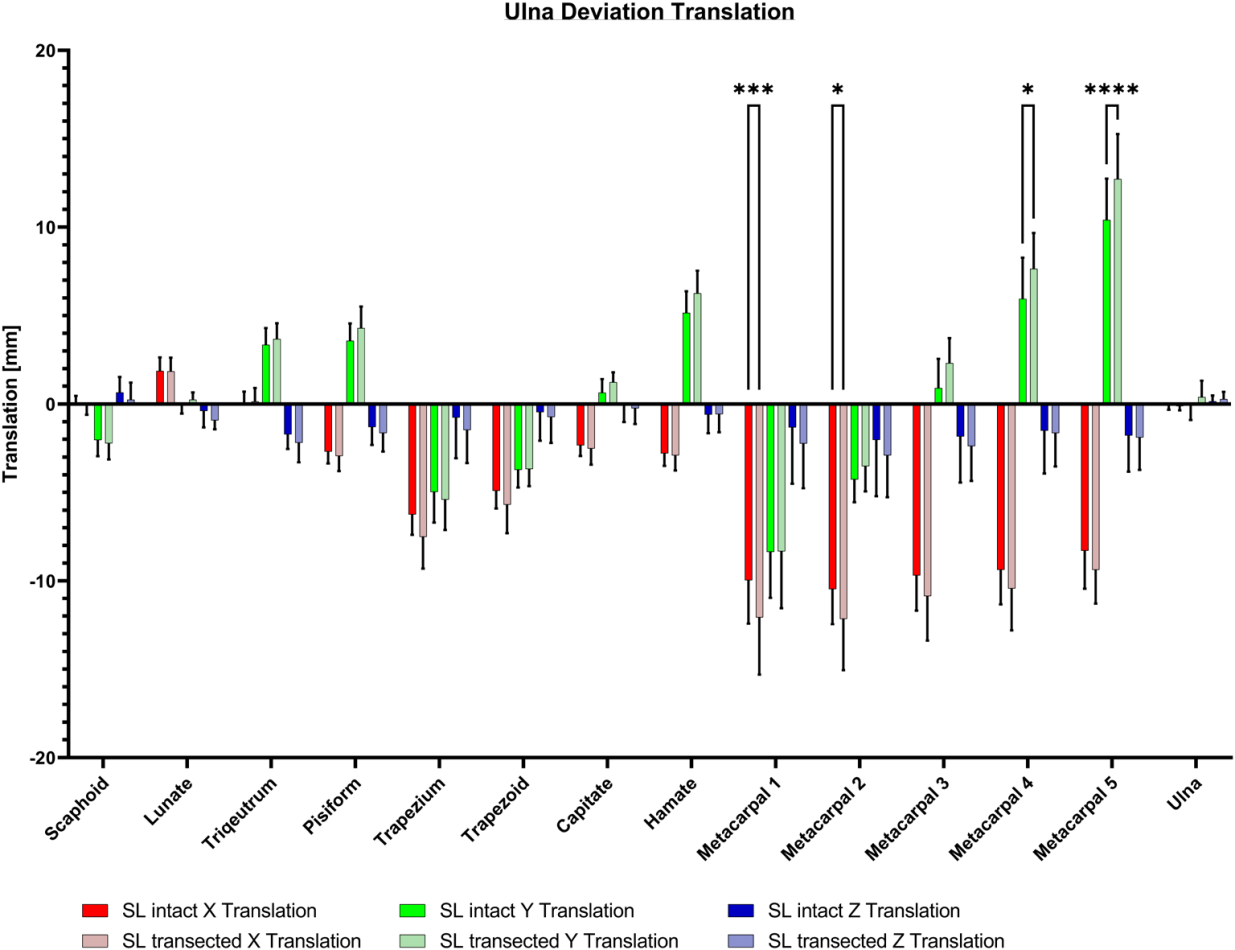
The results from the translation analysis during ulna deviation revealed no significant effect on carpal bones after SL transection. A significantly increased translation toward ulnar was seen for the first and second metacarpals following the transection of the SL ligament (*p* < 0.001–0.05), as well as a significant translation towards proximal for the fourth and fifth metacarpal after SL ligament transection (*p* < 0.05 and *p* < 0.0001). SL, scapholunate. *p < 0.05; ***p < 0.001; ****p < 0.0001.

**FIGURE 11:**
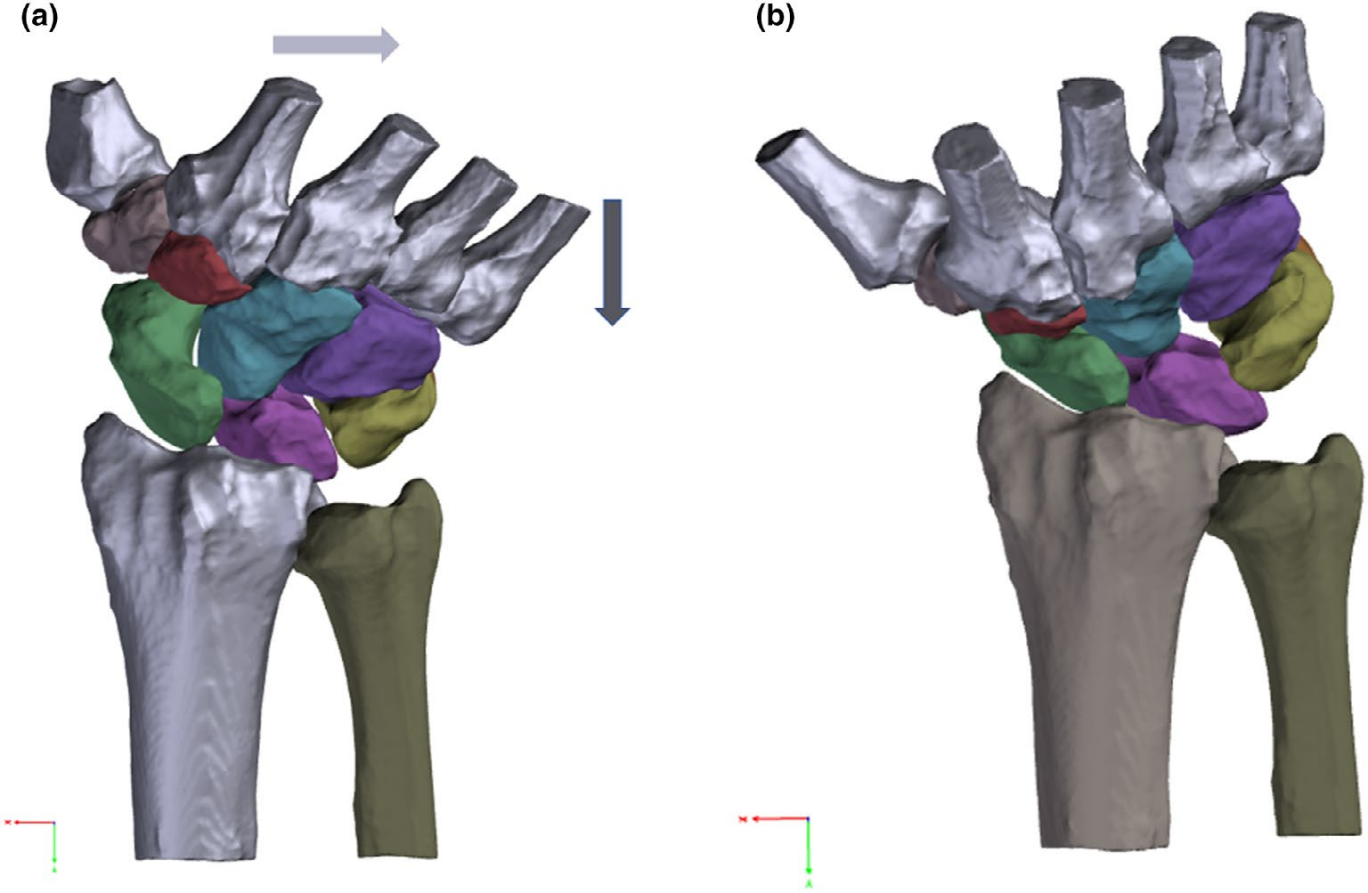
(a) Following SL‐ligament transection, a significant increase in the translation toward ulna of metacarpal 1 and 2 was observed during ulna deviation (light grey straight arrow). Additionally, a significant motion toward proximal was evident for metacarpal 4 and 5 (dark grey straight arrow). (b) Conversely, during radial deviation, no significant motions were detected for any bones, neither in rotation nor in translation analysis. SL, scapholunate.

The translation analysis showed no significant effect on carpal bones after SL ligament transection during ulna deviation. Further analysis of the *X*‐axis demonstrated statistical significance for translation toward ulnar for the first and second metacarpals after SL ligament transection (*p* < 0.001 and *p* < 0.05) (see Figure 10). Regarding the *Y*‐axis results, there is a contrasting motion among the metacarpal bones. The first two metacarpals exhibit a motion towards distal, while the third, fourth and fifth metacarpals translate towards proximal, showing a statistically significant increase of motion towards proximal after the transection of the SL ligament for the fourth and fifth metacarpal bone (*p* < 0.05 and *p* < 0.0001) (see Figure 11).

### 3.4 Radial deviation

In the rotation analysis during radial deviation, there is no significant impact neither on the proximal row nor on the distal row. During radial deviation, the most substantial rotation was observed in the *Z*‐axis. Once again, the results indicated that as the bones are located more distally, there is a greater degree of rotation. Interestingly, the first and second metacarpals exhibited a lesser extent of motion compared to the bones of the distal row.

The findings from the rotation analysis during radial deviation align with the results of the translation investigation. After SL ligament transection, there is no significant effect on the carpal bones during radial deviation in the translation analysis (see Figure 11). It is observed that the more distal the bones are located, the more pronounced the results, particularly in the *X*‐axis towards radial and in the *Z*‐axis towards dorsal, with the exception of the metacarpal 1, translating towards palmar.

In the *Y*‐axis analysis, a distinctive pattern emerged, revealing contrasting motion among the proximal row, the distal row and the metacarpals. The more radially located bones exhibited a pronounced translation towards the proximal direction, with the metacarpal bones showing the highest extent. However, none of these results reached statistical significance.

## 4 DISCUSSION

The SL ligament complex is a crucial component of the wrist's intricate ligamentous network (Pappou et al., 2013; Short et al., 2009). Understanding the SL ligament's role in wrist stability and its consequences upon transection are pivotal for diagnosing and treating wrist injuries and related conditions. The study findings support the critical role of the SL ligament in maintaining carpal stability during wrist movements. SL ligament lesions cause carpal structure displacement, SL interval dissociation, scaphoid flexion and lunate dorsal tilt (Rajan & Day, 2015b; White & Rollick, 2015). Wrist kinematics vary based on SL ligament injury severity (Buck‐Gramcko, 1985; Stromps et al., 2018; Taleisnik, 1980).

In the overall analysis the greatest motion extent was observed for the rotation in flexion and extension of the wrist, followed by the results for rotation in the ulnar and radial deviation, aligning with the results of earlier studies (Akhbari et al., 2019; Kobayashi et al., 1997; Li et al., 2022; Moojen et al., 2003; Short et al., 2002). Furthermore, translation results were most pronounced for more distally located bones, which can be attributed to the location of the centre of the coordinate system in the radius.

In a healthy wrist, the SL ligament prevents excessive scaphoid flexion and lunate extension, maintaining joint integrity and biomechanical efficiency. Isolated SL ligament transection increased carpal bone rotation and translation with a certain variation in between the different analysed motions. As could be assumed, there was an effect on the rotation and translation of the scaphoid, which is consistent with previously described results showing that the scaphoid is more flexed toward palmar, radially rotated and translates radial after SL ligament transection (Omori et al., 2013; Ruby et al., 1987; Short et al., 2002; Waters et al., 2016; Werner & Short, 2018). Omori et al. (2013) found a 7° radial rotation of the scaphoid with SL ligament transection in vivo, while Werner et al. (2011) observed less than 4° in cadavers. Our results showed similar results for the direction of motion with a significant increase for rotation during the flexion of 11.97° in comparison to the previously described 9° by Waters et al. (2016).

The previously described relatively minor impact on the rotation and translation of the lunate, also seen in our results, in contrast to the scaphoid, may be attributed to the lunate's inherent stability (Brinkhorst et al., 2021; Rainbow et al., 2013; Waters et al., 2016). This stability arises from its firm anchorage to the radius through the short and long radiolunate ligaments, in contrast to the scaphoid, which is more prone to distal displacement often facilitated by the scaphotrapezoidal and scaphocapitate ligaments (Andersson & Garcia‐Elias, 2013). Furthermore, these results can be rationalized by the anatomically strict fixation of the lunate, primarily attributed to the lunotriquetral intrinsic c‐shaped ligament and secondarily to the extrinsic ulnolunate ligament. These findings are in concordance with the observations made by Short et al. (1995) who noted increased scaphoid flexion, scaphoid pronation and lunate extension in cadaver forearms following the transection of the SL ligament. Likewise, Johnson et al. (2013) in their surface‐based analysis, observed an increased displacement of the scaphoid, whereas the lunate shifted toward the centre of the lunate fossa within the radiocarpal joint. These findings substantiate the hypothesis that the primary development of degenerative wrist arthritis is largely driven by increased flexion mobility of the scaphoid, a phenomenon we were also able to quantify. Furthermore, these rotational changes and their effect on the biomechanics of the wrist lead to patient discomfort and over time to wrist instabilities and arthritis (Kamal et al., 2016; Wayne & Tremols, 2019; Wessel & Wolfe, 2023). However, the precise clinical implications remain uncertain, primarily because our study, as well as most of the aforementioned studies, solely involved the examination of cadaver specimens without assessing the symptoms in actual patients.

Thus far, data on wrist bone motion after SL ligament transection in ulnar and radial deviation, particularly considering single carpal rows, remain limited. Our rotation analysis revealed only barely noticeable changes for the distal carpal row and the metacarpals after SL ligament transection, with no discernible impact on the proximal row during ulnar deviation. Moreover, no alterations were observed during radial deviation. Werner et al. (2011) reported that in the intact wrist, both the scaphoid and lunate exhibited statistically radial translation during ulnar deviation, which is only in accordance with our results for the lunate, as our results only showed a minor translation for the scaphoid. Importantly, the transection of the SL ligament did not have any impact on these observed results. Given the relatively minor carpal bone translations observed in general, our findings align with the conclusions drawn by Viegas et al. (1995) suggesting that translation is unlikely to occur unless multiple ligaments are transacted.

Regarding the carpal row theory, for which Kobayashi et al. (1997) already described a difference between the movement of the proximal and distal row in their analysis in 1997, we noted that the greatest extent of motion appeared between the radius and the bones of the proximal row, being followed by the midcarpal motion. However, our investigation showed no influence on the motion of the bones of the distal row after SL ligament transection. In the majority of the investigated motions the scaphoid took a special role of the bones from the proximal carpal row, which is similar to the findings of previous studies (Brinkhorst et al., 2021; Gill et al., 2022). Although, our findings suggest that the bones of the proximal carpal row achieve a unique degree of stability which is mitigated by the transection of the SL ligament.

In the account of our study, we are aware of the following limitations of the study. Our investigation presents solely the acute impact of wrist kinematics after an isolated SL ligament transection disregarding the other extrinsic and intrinsic carpal ligaments. Therefore, additional studies are needed to investigate the effect of a thorough transection of the further intrinsic as well as the extrinsic ligaments. However, the factor time is impossible to consider in thaw fresh‐frozen specimens as changes in the course of time will not affect the cadaver specimens. Moreover, the examination of cadaver specimens precludes the consideration of in vivo effects, such as active movements and muscle contraction, which play a pivotal role in the natural kinematics of the wrist. In vivo, muscle contractions and tendon forces contribute to the stabilization and movement of the carpal bones. These active forces can alter the alignment and interaction of the bones, potentially leading to different kinematic patterns than those observed in our static model. As such, while our findings provide valuable insights into the passive biomechanical behavior of the wrist following SL ligament transection, they may not fully represent the complexities of wrist motion under active conditions. Future studies incorporating dynamic testing and simulation of muscle forces are necessary to validate and extend our results. Despite these limitations, the consistency of our findings with previously reported data on passive wrist kinematics suggests that our results are credible, though they should be interpreted with caution in the context of active, in vivo wrist function.

Considering the treatment aspect of SL ligament injuries as hand surgeons continue their quest for the ideal treatment for SL tears, our data provide additional information about the importance of the different aspects of the SL ligament and the impact on the adjoining bones of the wrist. To what extent the biomechanics and the kinematic of the wrist are influenced after SL ligament treatment is subject to further investigations.

## 5 CONCLUSION

This study provides additional insight into the importance of the SL ligament concerning its role in wrist kinematics and SL stability. The results of this study add to the better understanding, that if the SL ligament is injured, failure of the ligament stabilization eventuate in an increased rotational motion of the scaphoid.

Furthermore, the analysis revealed that the isolated transection of the SL ligament had no discernible effect on the bones of the distal carpal row. The outcomes of our study lead to a better understanding of the various components necessary for enhancing the treatment of SL ligament injuries.

## AUTHOR CONTRIBUTIONS

**Dominik Promny**: Conceptualization; data curation; investigation; writing—original draft; Writing—review and editing. **Dominik Gill**: Data curation; formal analysis; investigation; methodology. **Stefan Lyer**: Formal analysis; writing—review and editing. **Christoph Alexiou**: Formal analysis; validation; writing—review and editing. **Thomas Buder**: Resources; writing—review and editing. **Winfried Neuhuber**: Resources; writing—review and editing. **Raymund E. Horch**: Formal analysis; funding acquisition; project administration; resources; supervision; validation; writing—review and editing. **Andreas Arkudas**: Conceptualization; Data curation; formal analysis; funding acquisition; investigation; methodology; project administration; resources; supervision; validation; writing—review and editing.

## FUNDING INFORMATION

This study was supported by the *Manfred Roth Foundation*, Fürth, Germany and the *Forschungsstiftung Medizin* at the Universitätsklinikum Erlangen, Germany.

## CONFLICT OF INTEREST STATEMENT

The authors declare that they have no known competing financial interests or personal relationships that could have appeared to influence the work presented in this manuscript. There are no conflicts of interest to declare.

## ETHICS STATEMENT

The study was carried out on fresh‐frozen anatomical specimens under the Anatomy Institute's body donation program for didactic, research and postgraduate training purposes. All donors gave written consent to the use of their bodies for scientific purposes in their donation document.

## Data Availability

The data that support the findings of this study are available on request from the corresponding author. The data are not publicly available due to privacy or ethical restrictions.
